# Understanding the vaginal microbiome among women with different genotypes of human papillomavirus infection in remote Andaman islands

**DOI:** 10.3389/fcimb.2024.1486166

**Published:** 2025-01-15

**Authors:** Rehnuma Parvez, Santhiya Vijayakumar, Alwin Vins, Sudha Ramaiah, Anand Anbarasu, Lipika Biswas, Nisha Beniwal, Harpreet Kaur, Nagarajan Muruganandam

**Affiliations:** ^1^ Indian Council of Medical Research - Regional Medical Research Centre, Port Blair, Andaman and Nicobar Islands, India; ^2^ Department of Biosciences, School of Biosciences and Technology (SBST), Vellore Institute of Technology (VIT), Vellore, India; ^3^ Department of Biotechnology, School of Biosciences and Technology (SBST), Vellore Institute of Technology (VIT), Vellore, India; ^4^ Division of Communicable Diseases, Indian Council of Medical Research - Head Quarters, New Delhi, India

**Keywords:** vaginal microbiome, human papillomavirus, South Andaman, women, symptomatic

## Abstract

**Background:**

Human papillomavirus (HPV) is a viral infection, and its acquisition and persistence are significantly influenced by the vaginal microbiota. Understanding and comparing the vaginal microbiome of HPV infected women in Andaman and Nicobar Islands is crucial.

**Methods:**

The study involved collecting vaginal swabs and extracting DNA using the QIAamp DNA Minikit. The DNA was then subjected to PCR amplification to confirm HPV infection. illumina NovaSeq 6000 platform was utilized to perform sequencing utilizing 2 x 250 paired end chemistry. Taxonomic analysis was performed and Bacterial abundance plots were generated and samples were grouped based on demographic parameters, pap test diagnosis, and genotypes. To assess diversity, samples were rarefied to 49,000 sequence reads per sample, and alpha and beta diversity metrics were calculated.

**Results:**

The study analyzed the presence of 21 assigned phyla, with Firmicutes, Actinobacteria, Bacteriodetes, and Proteobacteria emerging as the predominant taxa. At the genus level, *Lactobacillus* and *Gardnerella* dominated across all samples. *Gardnerella* was significantly more abundant in HPV-positive (22.40%) compared to HPV-negative samples (10.04%). Symptomatic group of HPV-positive samples had *Gardnerella*, and unclassified Coriobacteriaceae being dominant. In terms of bacterial diversity, the study found statistically significant association when comprising individuals aged 21 to 30 years to those aged 31 to 40 years.

**Conclusion:**

Most research concluded that exposure to HPV can boost bacterial diversity in vagina compared to healthy women, increasing the risk of cervical cancer development. Current study highlights the importance of vaginal microbiome associated with high and low risk HPV, various age group as well as the symptomatic and asymptomatic cases of HPV infected women in South Andaman.

## Introduction

Throughout a woman’s life, the vaginal microbiota undergoes changes, making it a complex and spontaneous micro-ecosystem. *Lactobacillus* species are typically dominant here, helping to maintain acidity and prevent infections (Chen et al., 2021). Variations in the microbiota of vagina can lead to conditions like bacterial vaginosis or yeast infections. Hormones, sexual contact, and hygiene practices can affect the overall makeup of the vaginal microbiome (Holdcroft et al., 2023).

According to research, a balanced vaginal microbial community dominated by *Lactobacillus species* may protect against numerous sexually transmitted illnesses (Chee et al., 2020). An infection known as the human papillomavirus (HPV) is generally acquired through sexual interaction. Almost every sexually active person will become infected at some point in their lives, generally without symptoms (Garcia et al., 2024). HPV can be transmitted through vaginal, anal, or oral sexual activity with an individual carrying the virus (Garcia et al., 2024).

Differences in the vaginal microbiome, such as low in *Lactobacillus* loads or raise in the diversity of distinct bacteria, have been related to an elevated possibility of cervical dysplasia and HPV infection (Głowienka-Stodolak et al., 2024). Cervical intraepithelial neoplasia (CIN) and cervical cancer (CC) have been found to be associated with a complex interaction between the microbes that live in the vagina and HPV, which is important for HPV acquiring and retention (Wang et al., 2023).

Among the microbial diversity of vaginal mucosa, Firmicutes, Bacteroidetes, Proteobacteria, and Actinobacteria were the most often observed taxa. Patients with HPV positivity had an excess of Actinobacteria, Proteobacteria, and Bacteroides. *Lactobacilli iners*, *Lactobacilli jensenii*, and *Lactobacilli crispatus* were the dominant *Lactobacillusspecies* among HPV positive cases. The most frequently identified pathogens in HPV-positive individuals were *Staphylococcus species*, *Enterococcus species*, and *Gardnerella vaginalis* (Santella et al., 2022).

*L. crispatus* and *L. iners* were found in a variety of states range from asymptomatic to atypical cytology/dysplasia. *C. trachomatis*, and *Prevotella* spp. were more predominantly identified with high risk-HPV infection and aberrant cytology. Furthermore, *Fusobacterium* and *Sneathia* were found in all phases of cervical carcinoma linked to HR-HPV (Mancilla et al., 2024).

According to population-based research carried out in South Andaman, women were most frequently infected with HPV-16, a high-risk HPV strain. The precancerous lesions and HPV positive as well as high-risk HPV 16 had a substantial connection with carcinoma of cervix (Parvez et al., 2024). Furthermore, various types of high-risk and low-risk HPV was detected by molecular techniques (Parvez et al., 2023). Microbial diversity of vagina linked with various types of HPV is not well studied among the women population in Andaman and Nicobar Islands. Therefore, we aimed to understand and compare the vaginal microbiome of HPV infected women of this Island.

## Materials and methods

Between December 2018 and April 2022, community-focused cross-sectional research of married women in the age range of 18 to 59 who resided in both municipal and rural region of the South Andaman Island, was conducted (Parvez et al., 2024). Women who were pregnant, having menstrual periods, following childbirth, had just had a hysterectomy or cervix removal, or who refused to take part in the research were excluded.

### Sample collection

Vaginal swabs were collected using two sterile cotton-tipped swabs from the vaginal wall and dorsal fornix. Specimens were collected in phosphate-buffered saline containing tube and stored in -40°C for long term storage. Samples were centrifuged at 10,000 rpm for 10minutes and the QIAamp DNA Minikit (Qiagen, Germany) was used for Nucleic acid extraction in accord with the instructions of the manufacturer. Subsequently, the quality of extracted DNA was determined using a spectrophotometer at 260 nm wavelength.

### Informed consent

Samples were taken after each person submitted a written consent form. All individuals who submitted informed consent was given instructions never utilize lubricants, douches, or vaginal contraception for at least 48 hours prior to sample collection. Moreover, refrain from having sex the evening before sample collection.

### PCR assay for HPV

The L1 consensus gene was the target of PCR amplification, a standard procedure that was applied to all samples’ DNA in order to ensure the presence of HPV. The L1 PCR reaction was prepared of a total volume of 50 µl which consisted of 5µl of 10X Taq buffer, 1µl of 0.2 mM dNTPs, 1 µl of forward ‘5-GCMCAGGGWCATAAYAATGG-3’ and reverse primers 5’-CGTCCMAARGGAWACTGATC-3’, 3U of 0.7 µl Taq DNA polymerase, 36.3 µl of nuclease-free water, and 5.0µl of extracted DNA. For the L1 gene amplification, the following PCR temperature cycling parameters were used: a 5-minute initial denaturation at 95°C, followed by 45 cycles of denaturation at 95°C for 30 seconds, annealing at 54°C for 40 seconds, extension at 72°C for 40 seconds, and a 5-minute final extension at 72°C. The amplification products, which comprised roughly 450 bp, was visible following electrophoresis on a 1% agarose gel was considered as HPV positive (Parvez et al., 2023).

The DNA sequencing and analysis of L1 gene PCR was carried out by using Sanger Sequencing method to confirm the HPV types distributed in South Andaman (Parvez et al., 2023).

### Type specific PCR assay

The major genotypes of HR-HPV 16 and 18 were detected using a type-specific PCR test (Parvez et al., 2023). To confirm the presence of the HR-HPV16, PCR was performed using a 50 µl reaction volume, that included 5 µl of Taq10x buffer, 1 µl of 0.2mM dNTP mix, 1 µl each of forward 5’-AAGGCCAACTAAATGTCAC-3’ and reverse primers 5’-CTGCTTTTATACTAACCGG-3’, 1 µl of 3U Taq DNA polymerase, 36.3 µl of nuclease free water, and 5 µl of isolated DNA. The thermo cycling conditions for amplification of HPV16 gene were: initial denaturation at 94°C for 5 minutes, proceeded by 40 cycles of denaturation at 94°C for 1 minute, annealing at 55°C for 1 minute, extension at 72°C for 1 minute, and a final extension of 5 minutes at 72°C. The amplification products, which comprised roughly 217 bp, was visible following electrophoresis on a 1.5% agarose gel were considered as HR-HPV 16 positive (Parvez et al., 2023).

The HR-HPV18 gene PCR was carried out in a 50 µl final volume, with the following components: 5 µl of Taq10x buffer, 1 µl of 0.2mM dNTP mix, 1µl each of forward 5’-CCGAGCACGACAGGAGAGRCT-3’ and reverse primer 5’-TCGTTTTCTTCCTCTGAGTCGCTT-3’, 0.7 µl of 3U Taq DNA polymerase, 36.3 µl of nuclease-free water, and 5 µl of purified DNA. Following a 5-minute initial denaturation at 94°C, the PCR for HPV 18 underwent 45 cycles of denaturation at 95°C for 30 seconds, annealing at 56°C for 40 seconds, extension at 72°C for 1 minute, and a final 5-minute extension at 72°C. The amplification products, which comprised roughly 172 bp, was visible following electrophoresis on a 2% agarose gel were considered as HR-HPV 18 positive (Parvez et al., 2023).

### Sample selection for sequencing

Samples were categorized based on the following criteria: (a) HPV-positive and negative cases, (b) Symptomatic and asymptomatic distribution of HPV-positive cases, (c) Location-wise distribution of HPV-positive population, (d) Age-wise distribution of HPV-positive cases, (e) Pap test diagnosis of HPV-positive cases, and (f) Genotype-based distribution in HPV-positive samples.

### 16S rRNA high-throughput gene sequencing

The National Institute of Biomedical Genomics (NIBMG) obtained the chosen vaginal samples in order to perform amplicon-based sequencing of the 16S rRNA gene’s V3-V4 hyper-variable region. Universal barcoded sets of primers such as 175 F (5’ - CCTACGGGNGGCWGCAG - 3’) and 512 R (5’ - GACTACHVGGGTATCTAATCC - 3’) (McDonald et al., 2012) were used to amplify the V3-V4 hyper variable region of the 16S rRNA gene from extracted DNA.

A mixture of 10X PCR buffer, 0.5 mM Magnesium Sulfate (MgSO4), DNA Taq Polymerase, nuclease free water, and 0.2 mM dNTPs was added to 4 μl of microbial DNA, which had a concentration of more than 15ng/μl. The mixture was amplified using PCR with the following conditions: 35 cycles of reaction including denaturation at 94°C for 30 seconds, annealing at 55°C for 30 seconds, and extension at 68°C for one minute. After that, the temperature was raised to 68°C for duration of a one minute, and subsequently maintained at 10 degree Celsius until subsequent processing. AgencourtAMPure-XP (Beckman Coulter) paramagnetic beads were used to purify the amplified products comprised of 510 bp size following 1% agarose gel electrophoresis. The Nextera XT Index Kit (Illumina) was utilized for sample indexing, and the Qubit Fluorometer along with QubitTM dsDNA HS test Kit (Invitrogen) were used for DNA library quantification. Additionally, the length of the amplicons was assessed using the 2100 Bioanalyzer instrument. After merging the libraries, the illumina NovaSeq 6000 platform was utilized to perform sequencing utilizing 2 x 250 paired-end chemistry. The resulting raw data was examined using a taxonomy classification approach.

### Bioinformatics and statistical analysis

The de-multiplexed raw reads of 16S rRNA were imported into the QIIME2 software using a manifest file which contains sample IDs and file paths essential for importing sequence data (Bolyen et al., 2019). Sample metadata file detailing each sample’s characteristics, such as ID, subject, age, ethnicity, and others were imported into the QIIME2 microbiome analysis software for further downstream analyses. The Cutadapt plug-in was used to remove adapter sequences from the raw sequence data to improve data quality. The high-quality paired-end reads were then merged after the removal of low-quality and chimeric sequences using q2-dada2 plug-in (Callahan et al., 2016). Subsequently, the sequences were clustered into amplicon sequence variants (ASVs) considering a threshold of 99% similarity using vsearch plug-in. The taxonomic analysis was performed using q2-feature-classifier and the Greengenes classifier was used to assign the taxonomy to the ASVs (McDonald et al., 2012). The Greengenes classifier was trained and tested to include only V3-V4 region of 16S rRNA, excluding other hyper-variable regions. The NTC sample was excluded from the analysis. Bacterial abundance plots were generated, focusing on taxonomic levels ranging from higher (phylum) to lower (genus). Samples were grouped based on demographic parameters, pap test diagnosis and genotypes to elucidate bacterial abundance in each comparison.

To assess diversity, the samples were rarefied to 49,000 sequence reads per sample to ensure uniform coverage depth. Alpha diversity metrics (Shannon, Simpson, Chao1 and ace) were calculated to evaluate the richness and diversity within samples, while Kruskal-Wallis tests were utilized to assess significance. Furthermore, the un-weighted UniFrac distance and the Bray-Curtis distance were considered as beta diversity metrics to measure sample diversity. The permutation multivariate analysis of variance (PERMANOVA) test was performed for beta-diversity analysis. Statistical comparisons of individual taxa abundance across sample groups were conducted in STAMPv2.1.3 (Parks et al., 2014). Welch’s t-test was carried out to distinguish two cohorts, using DP: The confidence intervals for 95% can be calculated using Welch’s inverted approach. Multiple group comparisons were done using ANOVA testing, and then Tukey-Kramer *post-hoc* tests without multiple test correction were performed. Findings that had a p-value less than 0.05 were considered as statistically significant.

## Results

Overall, 58 samples were chosen for the 16S rRNA sequencing. Of these 62% were HPV positive group whereas remaining 38% were HPV negative group. Among the HPV positives, 57% were symptomatic cases of HPV; meanwhile, 39% were asymptomatic cases. In addition, 68% of the samples were from rural women infected with HPV and 32% each from the age group of 31 to 40 and 41 to 50 years. Similarly, 67% of High-riskHPV-16 positive samples were selected for the sequencing. However, 15% of women belonged to the other high-risk HPV group and 7% belonged to low-risk HPV group.

**Figure 1:**
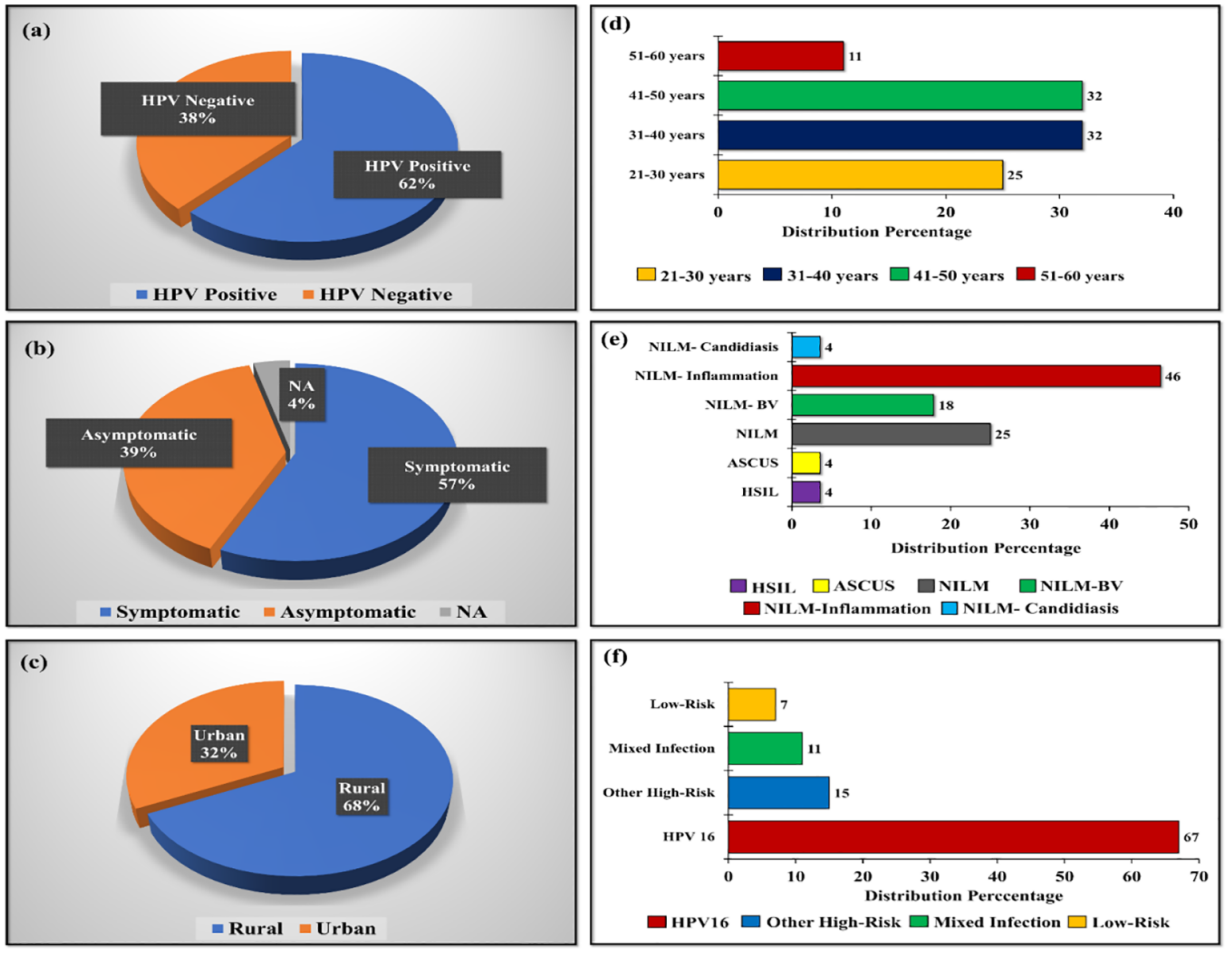
Distribution of samples categorized based on **(A)** HPV-positive and negative cases, **(B)** Symptomatic and asymptomatic distribution of HPV-positive population, **(C)** Location-wise distribution of HPV-positive population, **(D)** Age-wise distribution of HPV-positive population, **(E)** Pap test diagnosis of HPV-positive population, and **(F)** Genotype-based distribution in HPV-positive samples.

Forty-six percentage women selected were belonging to negative for intraepithelial lesion or malignancy (NILM)- inflammation category and 18% were belonged to NILM-Bacterial vaginosis. Apart from this, 4% of women were belonging to the group of NILM-Candidiasis. Samples categorized based on various criteria was shown in **Figure 1**.

**Figure 2:**
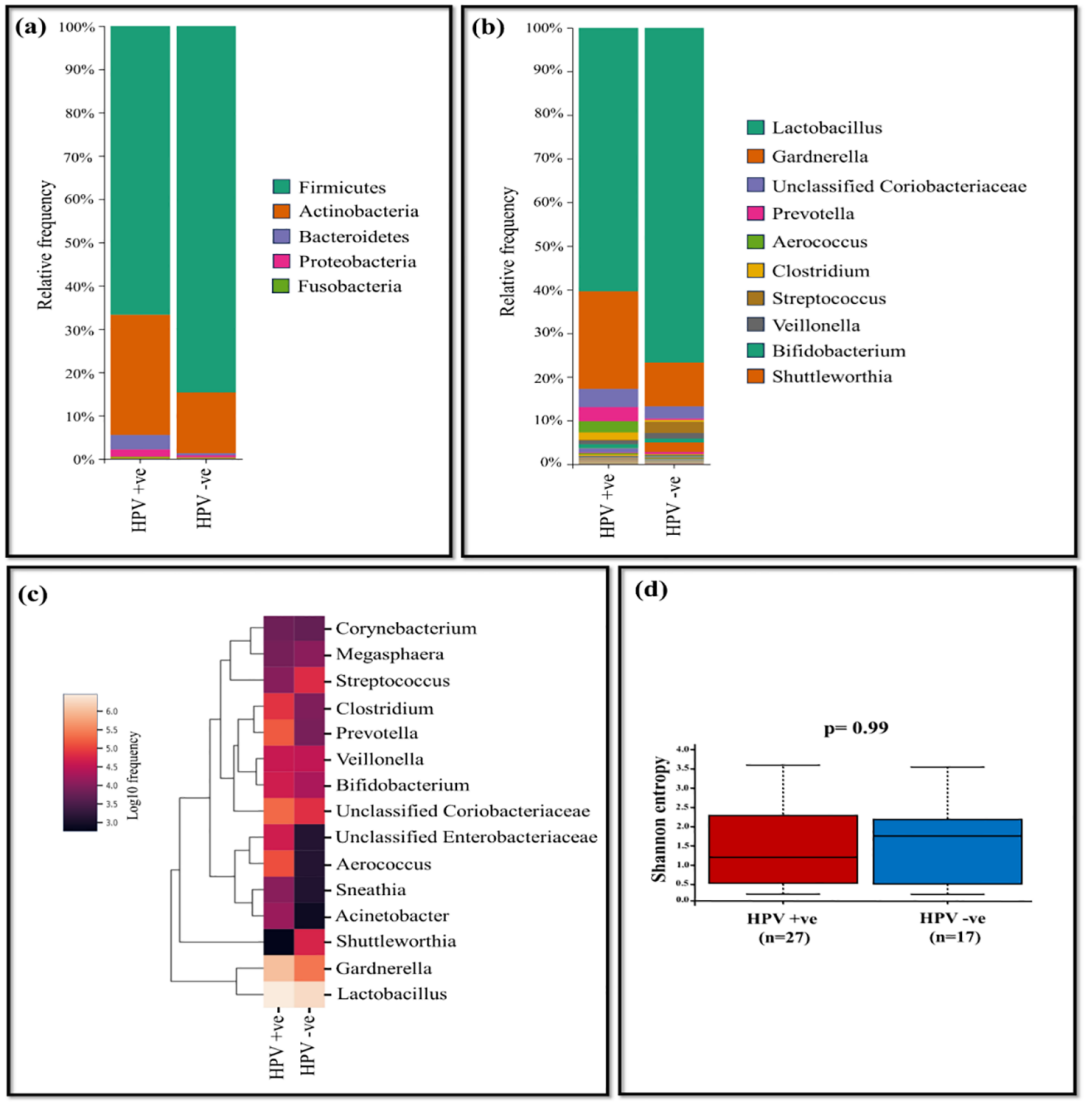
The vaginal microbiome comparison between HPV-positive and HPV-negative samples **(A)** The relative abundance level of various bacterial phyla **(B)** The relative abundance level of various bacterial genera **(C)** Taxonomic heatmap of top 15 abundant bacteria at genus level **(D)** Alpha diversity plot.

Our analysis identified the presence of 21 assigned phyla, with *Firmicutes*, *Actinobacteria*, *Bacteriodetes*, and *Proteobacteria* emerging as the prevalent taxa across all samples comprising a total genus of 99.46% and 99.63% for HPV-positive and negative samples, respectively. The remaining phyla exhibited negligible relative abundance. The most abundant phylum in both HPV-positive and negative samples was *Firmicutes* (66.69% vs 84.67%) followed by *Actinobacteria* (27.78% vs 14.04%), *Proteobacteria* (1.61% vs 0.47%) and *Bacteriodetes* (3.38% vs 0.44%) (**Figure 2A**).

Overall, at the genus level, *Lactobacillus* and *Gardnerella* dominated across all samples. *Lactobacillus* exhibited high relative abundance, accounting for 76.70% in HPV-negative samples and 60.35% for HPV-positive samples. *Gardnerella* was significantly more abundant in HPV-positive (22.40%) compared to HPV-negative samples (10.04%). Additionally, unclassified *Coriobacteriaceae* showed higher levels in HPV-positive samples at 4.16%, while in HPV-negative samples, it was 2.80% (**Supplementary Table 1**).

Other genera, such as *Prevotella* (3.23%), *Aerococcus* (2.61%), *Clostridium* (1.63%), demonstrated relatively higher levels in HPV-positive samples, while it was negligible in HPV-negative samples. Conversely, in HPV-negative samples, *Streptococcus* (2.55%), *Veillonella* (1.32%) and *Shuttleworthia* (2.18%) were dominant, with negligible relative abundance in HPV-positive samples. The tendency of decrease in the *Lactobacillus* genus in the HPV-positive group elevated the outgrowth of pathogenic genera such as *Gardnerella*, unclassified *Coriobacteriaceae*, *Prevotella*, and *Aerococcus* (**Figures 2B, C**). At the species level, *Lactobacillus* (*L*.) *iners* was more prevalent in HPV-positive samples (49.44%), whereas HPV-negative samples accounted for 44.70%. *L. helveticus* was dominant in HPV-negative samples (30.71%), whereas it was present at only 9.3% in HPV-positive samples. Shannon index assessed p= 0.99 showing no statistical difference in bacterial diversity between HPV-positive (n=28) and negative (n=17) samples (**Figure 2D**). According to Bray-Curtis distance and un-weighted UniFrac distance metrics, no significant differences were observed among the study groups.

**Figure 3:**
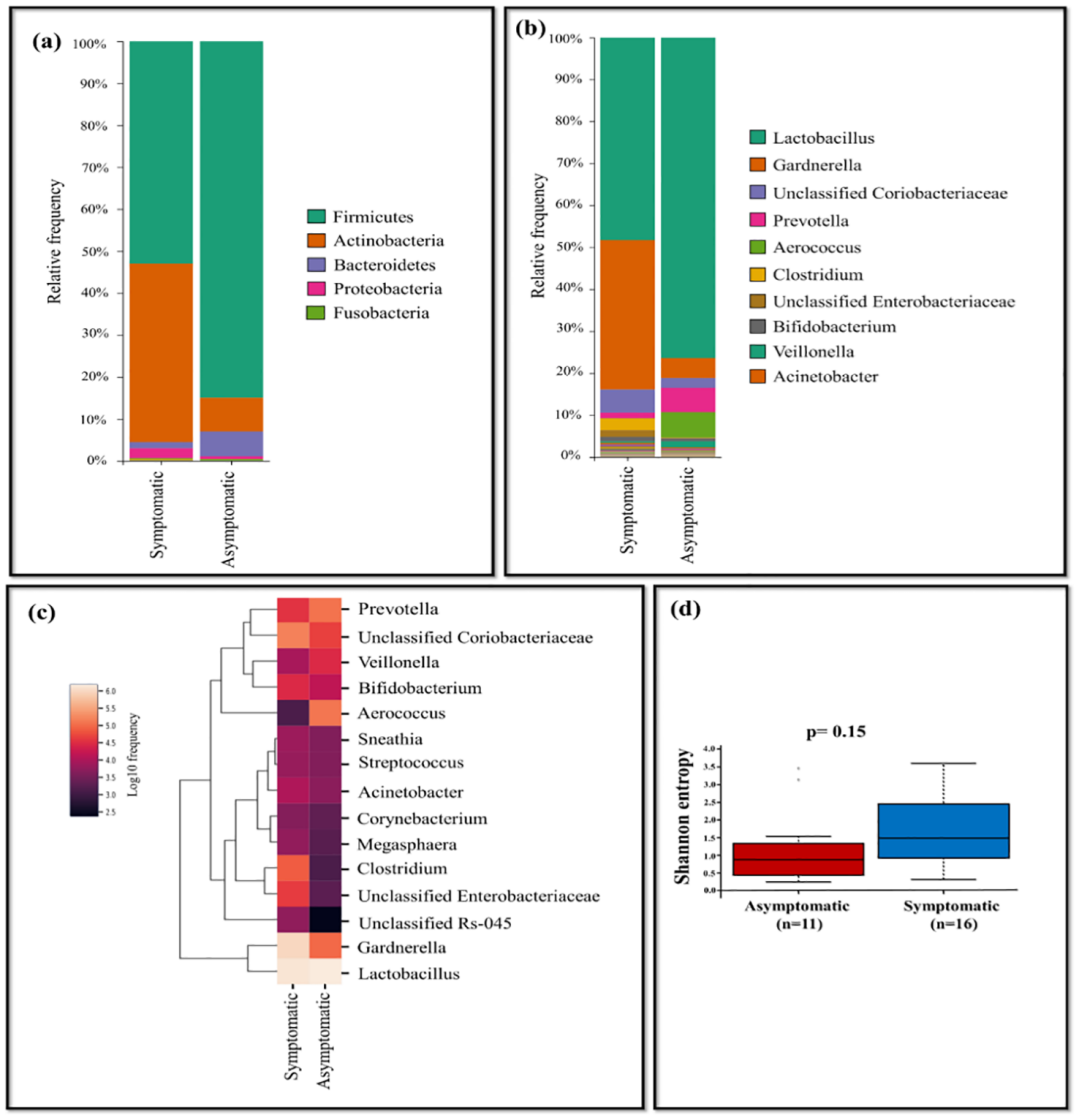
The vaginal microbiome comparison between symptomatic and asymptomatic group of HPV-positive samples **(A)** The relative abundance of different bacterial phyla **(B)** The relative abundance level of various bacteria genera **(C)** Taxonomic heatmap of top 15 abundant bacteria at genus level **(D)** Alpha diversity plot.

Further, HPV-positive samples were stratified based on metadata such as symptoms, location, age, pap test results, and genotypes to carry out comparison of taxonomic abundance. We observed differences in the microbiota of each comparison, providing insight into how these factors could influence the vaginal microbiome. The comparison between the symptomatic (n=16) and the asymptomatic group (n=11) of HPV-positive samples suggests, genus *Gardnerella* (35.59%), unclassified *Coriobacteriaceae* (5.53%), *Clostridium* (2.80%), *Bifidobacterium* (1.10%), unclassified *Enterobacteriaceae* (1.58%) were dominant in the symptomatic group of HPV-positive samples. In contrast, *Prevotella* (5.81%), *Aerococcus*(6.05%), *Veillonella* (1.44%) were dominant in the asymptomatic group of HPV-positive samples. *Acinetobacter* and *Sneathia* was present in negligible frequency in both symptomatic and asymptomatic groups (**Figures 3A-C**). The alpha diversity of samples from symptomatic and asymptomatic groups suggests diversity within the groups were not statistically significant (p=0.15) as the p value is not less than 0.05 (**Figure 3D**).

**Figure 4:**
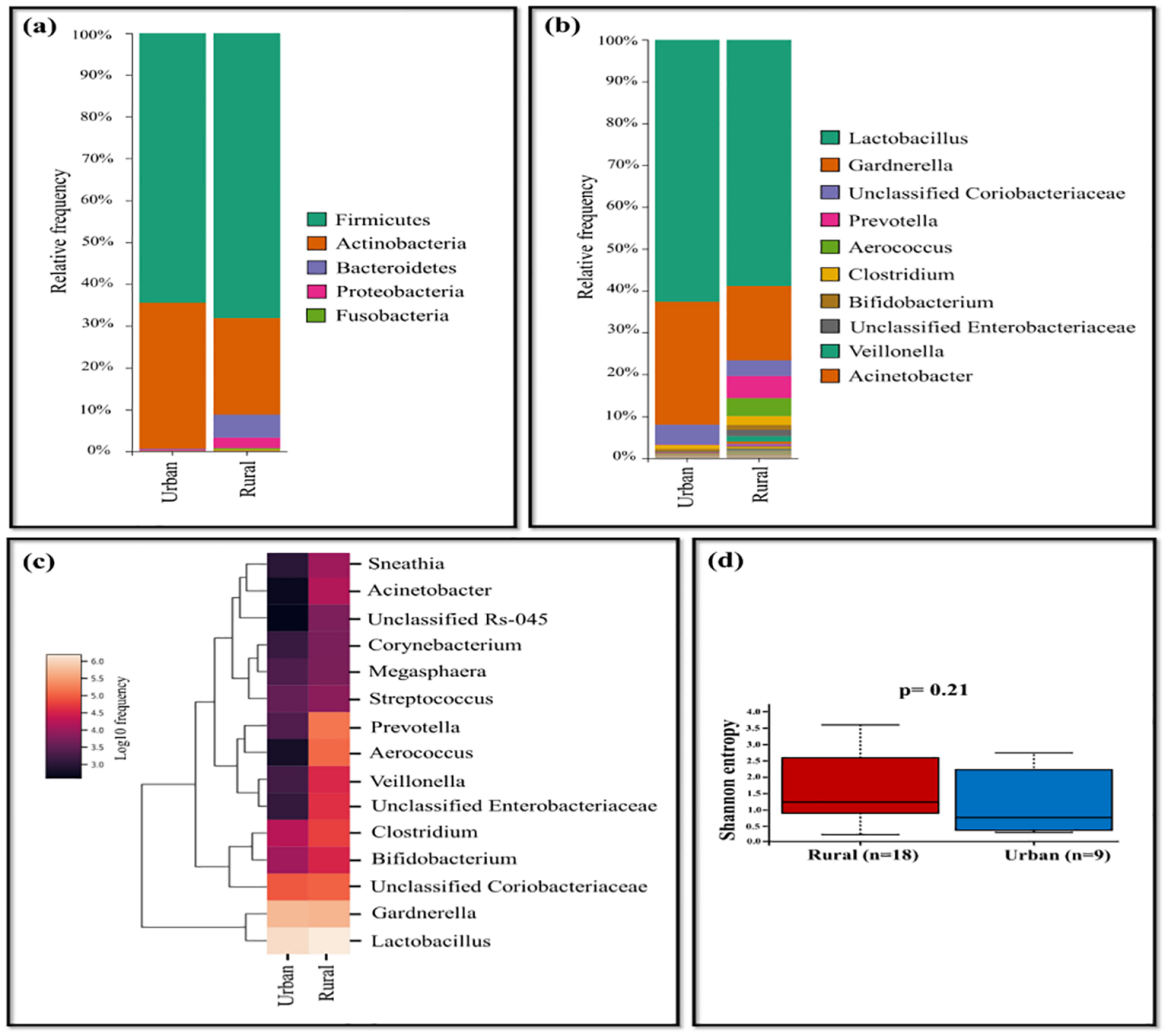
The vaginal microbiome comparison between urban and rural group of HPV-positive samples **(A)** The Relative abundance level of various bacterial phyla **(B)** The Relative abundance level of bacteria genera **(C)** Taxonomic heatmap of top 15 abundant bacteria at genus level **(D)** Alpha diversity plot.

The comparison of rural and urban groups revealed the dominance of *Lactobacillus* (62.64%), *Gardnerella* (29.38%), and unclassified *Coriobacteriaceae* (4.76%) in urban group of HPV-positive samples, while the rural group displayed prevalence of *Prevotella* (5.29%), *Aerococcus* (4.31%), *Clostridium* (2.10%), *Bifidobacterium* (1.17%), unclassified *Enterobacteriaceae* (1.52%), and *Veillonella* (1.28%). The occurrence of *Acinetobacter* and *Sneathia* were less frequent in both settings, with slightly higher levels observed in rural group (**Figures 4A-C**). Shannon diversity did not significantly differ between urban and rural group of HPV-positive samples (p=0.21) (**Figure 4D**).

**Figure 5:**
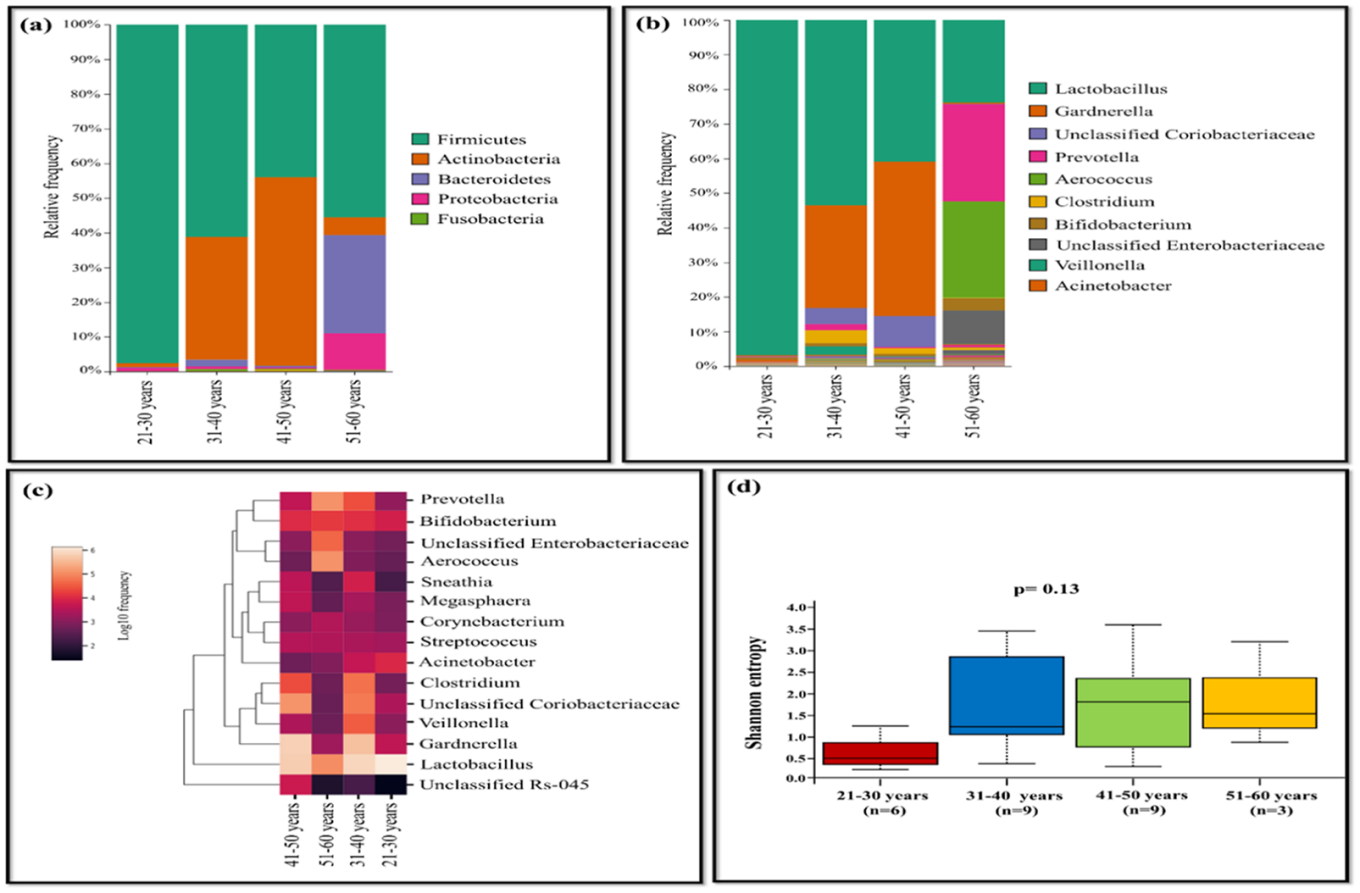
The vaginal microbiome comparison between different age group of HPV-positive samples **(A)** The Relative abundance level of bacterial phyla. **(B)** The Relative abundance level of bacterial genera **(C)** Taxonomic heatmap of top 15 abundant bacteria at genus level **(D)** Alpha diversity plot.

The age of the population tested positive for HPV ranged from 21 to 60 years. The samples were categorized into four age groups (21-30, 31-40, 41-50, and 51-60 years). *Lactobacillus* was highly abundant in age group of 21-30 years with 96.82%, while its prevalence was lowest in the age group of 51-60 years. In the age group of 41-50 years, *Gardnerella* (44.58%), unclassified *Coriobacteriaceae* (8.96%) dominated, displaying lower abundance in other groups. Samples of age group between 51-60 years showed dominance of *Prevotella* (28.04%), *Aerococcus* (27.88%), *Bifidobacterium* (3.52%), and unclassified *Enterobacteriaceae* (9.60%), whereas the other groups exhibited relatively less abundance (**Figures 5A–C**). Shannon diversity indicates statistically significant association (p=0.045) when comprising individuals aged 21-30 years to those aged 31-40 years, while other groups exhibited less statistical difference in terms of bacterial diversity (**Figure 5D**).

**Figure 6:**
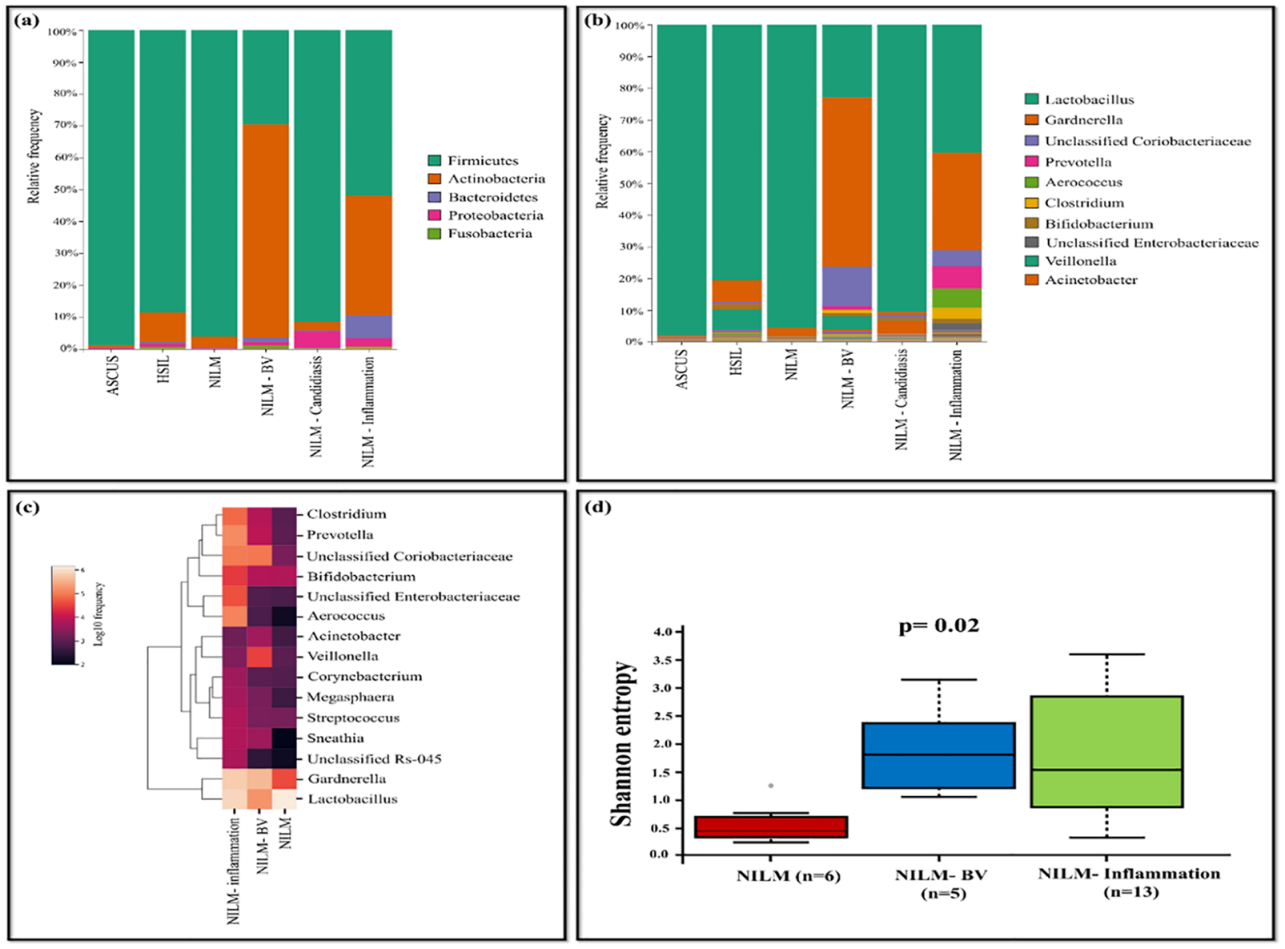
The vaginal microbiome comparison between groups based on pap-test results of HPV-positive samples **(A)** The Relative abundance level of bacterial phyla **(B)** Relative abundance level of bacterial genera **(C)** Taxonomic heatmap of top 15 abundant bacteria at genus level **(D)** Alpha diversity plot.

The HPV-positive samples were categorized based on pap-test results as follows: NILM (n=7), NILM-bacterial vaginosis (BV) (n=5), NILM-inflammation (n=13), NILM-candidiasis (n=1), Atypical squamous cells of undetermined significance (ASC-US) (n=1), High-grade squamous intraepithelial lesion (HSIL) (n=1). *Lactobacillus* (95.55%) was observed to be dominant in NILM group while it was comparatively less in other groups such as NILM-BV (22.94%) and NILM-inflammation (40.41%). *Lactobacillus* was less prevalent in the NILM group and an increased dominance of *Gardnerella* (53.36%) and unclassified *Coriobacteriaceae* (12.42%) was observed. The NILM-inflammation group exhibited dominance of *Prevotella* (7.07%), *Aerococcus* (6.06%), *Clostridium* (3.41%) and unclassified *Enterobacteriaceae* (2.13%) (**Figures 6A-C**). The analysis of Shannon diversity index revealed statistically significant difference between the groups NILM and NILM-BV (p=0.017). The NILM-BV group displayed higher bacterial diversity, as indicated by a higher Shannon diversity index compared to the NILM group. Additionally, a significant difference between NILM and NILM-inflammation group was detected (p=0.017) (**Figure 6D**).

**Figure 7:**
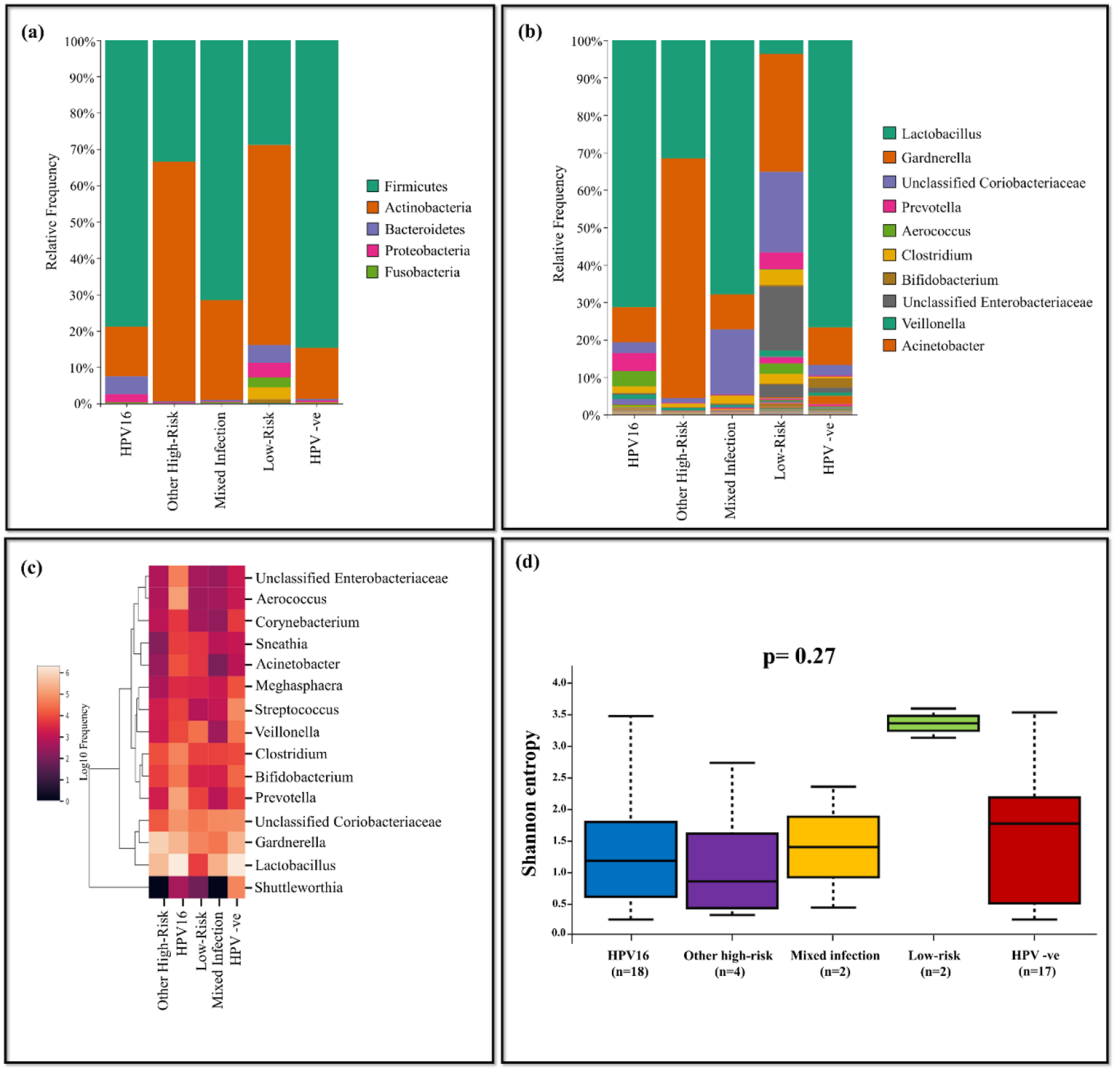
The vaginal microbiome comparison between groups based on genotypes of HPV-positive with HPV-negative population **(A)** The Relative abundance level of bacterialphyla **(B)** The Relative abundance level of bacterial genera **(C)** Taxonomic heatmap of top 15 abundant bacteria at genus level **(D)** Alpha diversity plot.

The HPV-positives were categorized based on their genotypes as follows: HPV16 (n=18), other high-risk (n=4), mixed infection (n=2), low-risk (n=2) and compared with HPV-negative group (n=17). *Lactobacillus* was predominant in HPV-negative group with bacterial abundance of 76.70%, while the least was for the low-risk group (3.66%). *Gardnerella* was highly abundant in other high-risk group with 64.02% among all the comparison groups. *Prevotella* (4.77%) and *Aerococcus* (4.08%) were dominant in HPV16 group, while *Streptococcus* (2.55%) and *Veillonella* (1.32%) were dominant in HPV-negative group. *Shuttleworthia* was predominant in HPV-negative group with 2.18%, while the abundance of the same in other groups was very negligible. The HPV16 group exhibited the high prevalence of unclassified *Enterobacteriaceae* with 1.45%, while it was comparatively less in other groups. Low-risk group displayed dominance of *Megasphaera* (1.55%), *Acinetobacter* (2.79%), and *Sneathia* (2.62%), while these were less in other groups (**Figures 7A-C**). The Shannon diversity index revealed statistically significant difference between HPV-negative and low-risk group with a p-value of 0.03. Additionally, a p-value of 0.04 was observed between HPV16 and low-risk group indicating significant difference (**Figure 7D**).

## Discussion

Our study applied established methodologies for analyzing the vaginal microbiome, utilizing QIIME2 to differentiate between HPV-positive and HPV-negative samples. The results indicate elevated bacterial diversity across cohorts, accompanied by a decrease in *Lactobacillus* species. The comparisons were based on HPV-positive versus negative samples, genotypes of HPV-positive versus HPV-negative samples, as well as symptomatic versus asymptomatic, rural versus urban, different age groups and pap test results among HPV-positive samples. As a metabolic byproduct, lactobacillus creates lactic acid, which contributes to the maintenance of a pH balance in the vaginal mucosa and inhibits the development of detrimental bacteria (Lin et al., 2021; Stoian et al., 2023). For the protection of reproductive health, the vaginal microbiota is essential (Holdcroft et al., 2023).

One important aspect concerning the vaginal microbiota is the predominance of *Lactobacillus* species (Chee et al., 2020; Ashok et al., 2024). In contrast to HPV-positive samples, the total proportion of *Lactobacillus species* was higher in HPV-negative samples; *L. iners* and *L. helveticus* dominated both. The dominance of *L. helveticus* was revealed in samples without HPV-positives (30.70%), while *L. iners* observed as the dominant species in HPV-positive samples (49.44%) compared to HPV-negative samples (44.70%).The roles of each *Lactobacillus* species differ from one another, and *L. iners*, the smallest *Lactobacillus* is reported to be the prevalent species in vaginal microbiome during transition from normal to abnormal states, linking to increased occurrence of HPV, human immunodeficiency virus and herpes simplex virus-2 (Ravel et al., 2011). A study conducted on *G. vaginalis*-induced BV mice demonstrated the probiotic benefits of *L. helveticus* HY7801, which helped in improving the vaginal health of the mice by reducing the levels of *G. vaginalis* and pro-inflammatory cytokines (Kim et al., 2023).

Our study also highlights a correlation observed in the vaginal microbiome between the asymptomatic group (n=11) and the NILM group (n=7) of HPV-positive samples. In both groups, the predominant genus was *Lactobacillus*, while *Gardnerella* and unclassified *Coriobacteriaceae* were less prevalent. This insight into the vaginal microbiome suggests that the high dominance of *Lactobacillus* hinders the prevalence of other pathogenic bacteria. In contrast, when comparing the symptomatic group with groups such as NILM-BV and NILM-inflammation, a notable increase in abundance was observed for *Gardnerella.* The lower abundance of *Lactobacillus* in these groups was associated with an increased propensity for the rise of pathogenic bacteria.

Furthermore, this study supports earlier findings that *Lactobacillus* levels decrease with age, offering insights specific to the population from the remote Andaman Islands (Zeber-Lubecka et al., 2022). *Lactobacillus* was dominant in the younger population (aged between 21 and 30 years), but significantly reduced in the elderly population (aged between 51 and 60years). This decline in *Lactobacillus* species has led to the overgrowth of harmful genera such as *Gardnerella*, unclassified *Coriobacteriaceae*, *Prevotella*, *Aerococcus*, and Unclassified *Enterobacteriaceae* in population aged between 41 and 60 years. Similar findings have been reported in another study where lack of estrogen due to ageing is associated with reduced *Lactobacillus* and a raise in pathogenic anaerobes in the vagina (Zeber-Lubecka et al., 2022).

Apart from age, the distribution of genera in the microbial diversity of vagina shows differences with the population from various locations i.e., rural and urban regions (Frąszczak et al., 2023). *Lactobacillus species* are more predominant in densely populated town in contrast to rural regions. This difference in the *Lactobacillus* abundance can be correlated with the overgrowth of harmful bacteria such as *Prevotella*, *Aerococcus*, *Veillonella*, *Acinetobacter* and *Sneathia* in rural areas. In contrast, another study examining the distribution of LAB species based on rural versus urban region revealed a predominance of *L. acidophilus*, *L. fermentum*, *L. gasseri*, *L. rhamnosus*, *L. plantarum* in rural areas, with the exception of *L. delbrueckii* (Frąszczak et al., 2023).

Women with HR HPV infections had more diverse microbiotas than those with HPV-33 or other virus genotypes, according to a study. Furthermore, women with numerous HR HPV genotype infections had larger proportions of *Streptococcus anginosis*, *Lactobacillus jemsenii*, and *Sneathiaamnii* than did those with LR HPV infection (Liu et al., 2022). However, the distribution of bacterial genera among different genotype groups revealed that *Lactobacillus* present predominantly in HPV-negative samples compared to all other genotype groups. HPV16 positive samples showed predominance of pathogens such as *Prevotella* and *Aerococcus*. Notably, *Megasphaera*, *Acinetobacter*, *Sneathia*, *Clostridium* and *Bifidobacterium* were dominant in low-risk infection. In order to solve further questions, research on association of HPV genotypes and vaginal microbiota are required. They also offer a potential therapeutic target and low-cost options for future treatment approaches.

The present study suggests that samples of HPV-positive group exhibited low *Lactobacillus species* and increased presence of *Prevotella*, and *Gardnerella* compared to HPV-negative group. The observed dominance of *L. iners* in our study groups contributes to the existing evidence of bacterial dysbiosis, providing insights into its potential role in disease predisposition in this specific population (Ravel et al., 2011; Zheng et al., 2021). Analyses employing high-throughput genome sequencing approaches have found unique bacterial populations among the women with HPV infection. These new findings will help shape future clinical studies on the importance of vaginal microbiome in women’s’ health and illnesses.

## Data Availability

The datasets presented in this study can be found in online repositories. The names of the repository/repositories and accession number(s) can be found below: https://www.ncbi.nlm.nih.gov/ SRR31798762, SRR31798770, SRR31798767, SRR31798778, SRR31798774, SRR31798764, SRR31798780, SRR31798775, SRR31798773, SRR31798785, SRR31798777, SRR31798771, SRR31798765, SRR31798801, SRR31798799, SRR31798772, SRR31798788, SRR31798802, SRR31798760, SRR31798776, SRR31798794, SRR31798761, SRR31798782, SRR31798763, SRR31798800, SRR31798768, SRR31798793, SRR31798766, SRR31798790, SRR31798791, SRR31798781, SRR31798795, SRR31798786, SRR31798769, SRR31798787, SRR31798783, SRR31798804, SRR31798779, SRR31798803, SRR31798797, SRR31798792, SRR31798798, SRR31798796, SRR31798784, SRR31798789.

## References

[B1] Ashok G. Basu S. Priyamvada P. Anbarasu A. Chintala S. Ramaiah S. (2024). Coinfections in human papillomavirus associated cancers and prophylactic recommendations. Rev. Med. Virol. 34. doi: 10.1002/rmv.2524 38375992

[B2] Bolyen E. Rideout J. R. Dillon M. R. Bokulich N. A. Abnet C. C. Al-Ghalith G. A.. (2019). Reproducible, interactive, scalable and extensible microbiome data science using QIIME 2. Nat. Biotechnol. 37, 852–857. doi: 10.1038/s41587-019-0209-9 31341288 PMC7015180

[B3] Callahan B. J. McMurdie P. J. Rosen M. J. Han A. W. Johnson A. J. A. Holmes S. P. (2016). DADA2: High-resolution sample inference from Illumina amplicon data. Nat. Methods 13, 581–583. doi: 10.1038/nmeth.3869 27214047 PMC4927377

[B4] Chee W. J. Y. Chew S. Y. Than L. T. L. (2020). Vaginal microbiota and the potential of Lactobacillus derivatives in maintaining vaginal health. Microb. Cell Fact. 19, 203. doi: 10.1186/s12934-020-01464-4 33160356 PMC7648308

[B5] Chen X. Lu Y. Chen T. Li R. (2021). The female vaginal microbiome in health and bacterial vaginosis. Front. Cell. Infect. Microbiol. 11. doi: 10.3389/fcimb.2021.631972 PMC8058480 33898328

[B6] Frąszczak K. Barczyński B. Siwiec R. Kondracka A. Malm A. Kotarski J.. (2023). The analysis of Lactobacillus spp. distribution in the vaginal microbiota of Polish women with abnormal Pap smear result. Front. Microbiol. 14. doi: 10.3389/fmicb.2023.1257587 PMC10666048 38029074

[B7] Garcia M. R. Leslie S. W. Wray A. A. (2024). “Sexually transmitted infections,” in StatPearls (StatPearls Publishing, Treasure Island (FL). Available at: https://www.ncbi.nlm.nih.gov/books/NBK560808/. 32809643

[B8] Głowienka-Stodolak M. Bagí Nska-Drabiuk K. Szubert S. Hennig E. E. Horala A. Abrowska M. D.. (2024). Human papillomavirus infections and the role played by cervical and cervico-vaginal microbiota—Evidence from next-generation sequencing studies. Cancers 16, 399. doi: 10.3390/CANCERS16020399 38254888 PMC10814012

[B9] Holdcroft A. M. Ireland D. J. Payne M. S. (2023). The vaginal microbiome in health and disease—What role do common intimate hygiene practices play? Microorganisms 11, 298. doi: 10.3390/microorganisms11020298 36838262 PMC9959050

[B10] Kim J. Y. Moon E. C. Kim J. Y. Kim H. J. Heo K. Shim J. J.. (2023). Lactobacillus helveticus HY7801 ameliorates bacterial vaginosis by inhibiting biofilm formation and epithelial cell adhesion of Gardnerella vaginalis. Food Sci. Biotechnol. 32, 507–515. doi: 10.1007/S10068-022-01208-7/FIGURES/4 36911333 PMC9992491

[B11] Lin Y. P. Chen W. C. Cheng C. M. Shen C. J. (2021). Vaginal pH value for clinical diagnosis and treatment of common vaginitis. Diagnostics 11, 1996. doi: 10.3390/DIAGNOSTICS11111996 34829343 PMC8618584

[B12] Liu S. Li Y. Song Y. Wu X. Baloch Z. Xia X. (2022). The diversity of vaginal microbiome in women infected with single HPV and multiple genotype HPV infections in China. Front. Cell. Infect. Microbiol. 12. doi: 10.3389/fcimb.2022.642074 PMC9806233 36601309

[B13] Mancilla V. Jimenez N. R. Bishop N. S. Flores M. Herbst-Kralovetz M. M. (2024). The vaginal microbiota, human papillomavirus infection, and cervical carcinogenesis: A systematic review in the latina population. J. Epidemiol. Glob. Health 14, 480–497. doi: 10.1007/s44197-024-00201-z 38407720 PMC11176136

[B14] McDonald D. Price M. N. Goodrich J. Nawrocki E. P. Desantis T. Z. Probst A.. (2012). An improved Greengenes taxonomy with explicit ranks for ecological and evolutionary analyses of bacteria and archaea. ISME J. 6, 610–618. doi: 10.1038/ISMEJ.2011.139 22134646 PMC3280142

[B15] Parks D. H. Tyson G. W. Hugenholtz P. Beiko R. G. (2014). STAMP: statistical analysis of taxonomic and functional profiles. Bioinformatics 30, 3123. doi: 10.1093/BIOINFORMATICS/BTU494 25061070 PMC4609014

[B16] Parvez R. Vijayachari P. Saha M. K. Biswas L. Ramasamy J. Vins A.. (2023). Distribution of human papillomavirus genotypes among the women of south andaman island, India. Diagnostics 13, 2765. doi: 10.3390/diagnostics13172765 37685303 PMC10486394

[B17] Parvez R. Vijayachari P. Thiruvengadam K. Roy A. Saha M. K. Ramasamy J.. (2024). A population based study on human papillomavirus infection and associated risk factors among women of the remote South Andaman Island, India. BMC Womens. Health 24, 139. doi: 10.1186/s12905-024-02967-7 38395851 PMC10893608

[B18] Ravel J. Gajer P. Abdo Z. Schneider G. M. Koenig S. S. K. McCulle S. L.. (2011). Vaginal microbiome of reproductive-age women. Proc. Natl. Acad. Sci. U. S. A. 108, 4680–4687. doi: 10.1073/PNAS.1002611107/SUPPL_FILE/ST08.XLSX 20534435 PMC3063603

[B19] Santella B. Schettino M. T. Franci G. De Franciscis P. Colacurci N. Schiattarella A.. (2022). Microbiota and HPV: The role of viral infection on vaginal microbiota. J. Med. Virol. 94, 4478–4484. doi: 10.1002/JMV.27837 35527233 PMC9544303

[B20] Stoian I. L. Botezatu A. Fudulu A. Ilea C. G. Socolov D. G. (2023). Exploring Microbiota Diversity in Cervical Lesion Progression and HPV Infection through 16S rRNA Gene Metagenomic Sequencing. J. Clin. Med. 12, 4979. doi: 10.3390/JCM12154979/S1 37568379 PMC10420036

[B21] Wang Y. Thakur R. Shen Q. He Y. Chen C. (2023). Influences of vaginal microbiota on human papillomavirus infection and host immune regulation: What we have learned? Decod. Infect. Transm. 1, 100002. doi: 10.1016/j.dcit.2023.07.001

[B22] Zeber-Lubecka N. Kulecka M. Lindner B. Krynicki R. Paziewska A. Nowakowski A.. (2022). Increased diversity of a cervical microbiome associates with cervical cancer. Front. Oncol. 12. doi: 10.3389/fonc.2022.1005537 PMC9562559 36249017

[B23] Zheng N. Guo R. Wang J. Zhou W. Ling Z. (2021). Contribution of lactobacillus iners to vaginal health and diseases: A systematic review. Front. Cell. Infect. Microbiol. 11. doi: 10.3389/fcimb.2021.792787 PMC8645935 34881196

