# Supplementary material for: Understanding the vaginal microbiome among women with different genotypes of human papillomavirus infection in remote Andaman islands
Source: Front Cell Infect Microbiol. 2025 Jan 15;14:1486166. doi: 10.3389/fcimb.2024.1486166 (PMC11775162; doi:10.3389/fcimb.2024.1486166)
Supplement: Supplementary file 1 [file Table1.docx]

**Bacterial 16S rRNA Metagenomic Data Analysis of HPV positive and negative isolates**

**Genus level Bacterial abundance**

**1. HPV +ve VS HPV -ve**

| **SI.No** | **Genus** | **HPV +VE (n=28)** | **HPV -VE (n=17)** | **p-value** |
| --- | --- | --- | --- | --- |
| 1 | *Lactobacillus* | 60.352% | 76.702% | 0.686 |
| 2 | *Gardnerella* | 22.399% | 10.037% | 0.903 |
| 3 | Unclassified *Coriobacteriaceae* | 4.157% | 2.797% | 0.526 |
| 4 | *Prevotella* | 3.231% | 0.31% | 0.112 |
| 5 | *Aerococcus* | 2.608% | 0.05% | 0.328 |
| 6 | *Clostridium* | 1.628% | 0.358% | 0.198 |
| 7 | *Streptococcus* | 0.229% | 2.552% | 0.334 |
| 8 | *Veillonella* | 0.805% | 1.320% | 0.787 |
| 9 | *Bifidobacterium* | 0.946% | 0.850% | 0.555 |
| 10 | *Shuttleworthia* | 0.01% | 2.185% | 0.331 |
| 11 | *Enterobacteriaceae* | 0.944% | 0.05 % | 0.315 |
| 12 | *Megaspaera* | 0.162% | 0.455% | 0.402 |
| 13 | *Acinetobacter* | 0.333% | 0.033% | 0.120 |
| 14 | *Sneathia* | 0.240% | 0.049% | 0.040 |
| 15 | *Corynebacterium* | 0.138% | 0.212% | 0.498 |

**2. Symptomatic VS Asymptomatic**

| **SI.No** | **Genus** | **Symptomatic (n=16)** | **Asymptomatic (n=11)** | **p-value** |
| --- | --- | --- | --- | --- |
| 1 | *Lactobacillus* | 48.311% | 76.412% | 0.099 |
| 2 | *Gardnerella* | 35.591% | 4.752% | 0.031 |
| 3 | Unclassified *Coriobacteriaceae* | 5.526% | 2.332% | 0.193 |
| 4 | *Prevotella* | 1.318% | 5.809% | 0.463 |
| 5 | *Aerococcus* | 0.050% | 6.051% | 0.341 |
| 6 | *Clostridium* | 2.795% | 0.067% | 0.096 |
| 7 | *Bifidobacterium* | 1.099% | 0.735% | 0.401 |
| 8 | Unclassified *Enterobacteriaceae* | 1.579% | 0.093% | 0.339 |
| 9 | *Veillonella* | 0.332% | 1.444% | 0.452 |
| 10 | *Acinetobacter* | 0.389% | 0.257% | 0.853 |
| 11 | *Sneathia* | 0.265% | 0.208% | 0.621 |

**3. Rural VS Urban**

| **SI.No** | **Genus** | **Rural (n=19)** | **Urban (n=9)** | **p-value** |
| --- | --- | --- | --- | --- |
| 1 | *Lactobacillus* | 58.839% | 62.637% | 0.896 |
| 2 | *Gardnerella* | 17.783% | 29.376% | 0.471 |
| 3 | Unclassified *Coriobacteriaceae* | 3.760% | 4.757% | 0.604 |
| 4 | *Prevotella* | 5.291% | 0.11% | 0.080 |
| 5 | *Aerococcus* | 4.311% | 0.03% | 0.326 |
| 6 | *Clostridium* | 2.098% | 0.918% | 0.845 |
| 7 | *Bifidobacterium* | 1.175% | 0.598% | 0.178 |
| 8 | Unclassified *Enterobacteriaceae* | 1.524% | 0.067% | 0.326 |
| 9 | *Veillonella* | 1.277% | 0.092% | 0.224 |
| 10 | *Acinetobacter* | 0.536% | 0.02% | 0.114 |
| 11 | *Sneathia* | 0.364% | 0.053% | 0.069 |

**4. pap-test**

| **SI.No** | **Genus** | **NILM AND BV (n=5)** | **NILM and inflammation (n=13)** | **HSIL (n=1)** | **NILM and Candidiasis (n=1)** | **NILM (n=7)** | **ASC-US (n=1)** | **p-value** |
| --- | --- | --- | --- | --- | --- | --- | --- | --- |
| 1 | *Lactobacillus* | 22.939% | 40.411% | 80.581% | 90.566% | 95.549% | 97.845% | 0.005 (5.21e-3) |
| 2 | *Gardnerella* | 53.360% | 30.700% | 6.816% | 0.916% | 2.666% | 0.272% | 0.041 |
| 3 | Unclassified *Coriobacteriaceae* | 12.416% | 5.002% | 0.359% | 0.809% | 0.127% | 0.058% | 0.151 |
| 4 | *Prevotella* | 1.154% | 7.073% | 0.302% | 0.100% | 0.068% | 0.083% | 0.334 |
| 5 | *Aerococcus* | 0.089% | 6.063% | 0 | 0.046% | 0.010% | 0.060% | 0.651 |
| 6 | *Clostridium* | 0.958% | 3.406% | 0.050% | 0.011% | 0.062% | 0.120% | 0.406 |
| 7 | *Bifidobacterium* | 0.936% | 1.371% | 1.701% | 0.595% | 0.463% | 0.399% | 0.579 |
| 8 | Unclassified *Enterobacteriaceae* | 0.103% | 2.133% | 0.098% | 0.020% | 0.045% | 0.037% | 0.650 |
| 9 | *Veillonella* | 4.071% | 0.108% | 6.160% | 0.164% | 0.066% | 0.071% | 0.105 |
| 10 | *Acinetobacter* | 0.626% | 0.070% | 0.117% | 4.078% | 0.036% | 0 | 0.174 |
| 11 | *Sneathia* | 0.593% | 0.317% | 0.504% | 0.064% | 0.006% | 0 | 0.238 |

**5. Age**

| **SI.No** | **Genus** | **Between 21-30 (n=7)** | **Between 31-40 (n=9)** | **Between 41-50 (n=9)** | **Between 51-60 (n=3)** | **p-value** |
| --- | --- | --- | --- | --- | --- | --- |
| 1 | *Lactobacillus* | 96.822% | 53.541% | 40.913% | 23.824% | 0.030 |
| 2 | *Gardnerella* | 0.292% | 29.628% | 44.585% | 0.426% | 0.055 |
| 3 | Unclassified *Coriobacteriaceae* | 0.185% | 4.660% | 8.956% | 0.104% | 0.068 |
| 4 | *Prevotella* | 0.092% | 1.768% | 0.312% | 28.038% | 0.0001 (1.05e-4) |
| 5 | *Aerococcus* | 0.030% | 0.061% | 0.036% | 27.885% | 0.028 |
| 6 | *Clostridium* | 0.045% | 3.732% | 1.642% | 0.118% | 0.666 |
| 7 | *Bifidobacterium* | 0.487% | 0.856% | 0.719% | 3.516% | 0.039 |
| 8 | Unclassified *Enterobacteriaceae* | 0.042% | 0.076% | 0.078% | 9.600% | 0.028 |
| 9 | *Veillonella* | 0.081% | 2.405% | 0.187% | 0.113% | 0.407 |
| 10 | *Acinetobacter* | 0.649% | 0.341% | 0.039% | 0.210% | 0.741 |
| 11 | *Sneathia* | 0.01 | 0.504% | 0.270% | 0.059% | 0.384 |

**6. Serotypes**

| **SI.No** | **Genus** | **HPV 16 (n=18)** | **Low risk (n=2)** | **Mixed infection (n=3)** | **Other high risk (n=4)** | **HPV -ve (n=17)** | **p-value** |
| --- | --- | --- | --- | --- | --- | --- | --- |
| 1 | *Lactobacillus* | 71.307% | 3.659% | 67.873% | 31.596% | 76.702% | 0.178 |
| 2 | *Gardnerella* | 9.329% | 31.530% | 9.365% | 64.028% | 10.037% | 0.019 |
| 3 | Unclassified *Coriobacteriaceae* | 2.923% | 21.466% | 17.392% | 1.209% | 2.797% | 0.024 |
| 4 | *Prevotella* | 4.771% | 4.487% | 0.222% | 0.172% | 0.316% | 0.507 |
| 5 | *Aerococcus* | 4.080% | 0.200% | 0.108% | 0.053% | 0.053% | 0.858 |
| 6 | *Clostridium* | 1.786% | 4.073% | 2.011% | 0.903% | 0.358% | 0.710 |
| 7 | *Streptococcus* | 0.232% | 0.409% | 0.340% | 0.169% | 2.552% | 0.828 |
| 8 | *Veillonella* | 0.282% | 17.114% | 0.094% | 0.135% | 1.320% | 0.020 |
| 9 | *Bifidobacterium* | 1.083% | 1.517% | 0.616% | 0.542% | 0.850% | 0.932 |
| 10 | *Shuttleworthia* | 0.01% | 0.04% | 0 | 0 | 2.185% | 0.824 |
| 11 | Unclassified *Enterobacteriaceae* | 1.446% | 0.260% | 0.082% | 0.050% | 0.054% | 0.853 |
| 12 | Megasphaera | 0.102% | 1.552% | 0.399% | 0.048% | 0.455% | 0.375 |
| 13 | Acinetobacter | 0.363% | 2.793% | 0.02% | 0.02% | 0.033% | 0.016 |
| 14 | *Sneathia* | 0.206% | 2.620% | 0.228% | 0.01% | 0.04% | <0.0001 |
| 15 | *Corynebacterium* | 0.163% | 0.245% | 0.060% | 0.082% | 0.212% | 0.907 |
